# Consciousness in Solitude: Is Social Interaction Really a Necessary Condition?

**DOI:** 10.3389/fpsyg.2021.630922

**Published:** 2021-02-16

**Authors:** Sepehrdad Rahimian

**Affiliations:** Department of Psychology, National Research University Higher School of Economics, Moscow, Russia

**Keywords:** self, SOMA, attention-schema theory, subjective experience, consciousness

## Introduction

Recently, Cleeremans et al. (2020a) presented their Self-Organizing metarepresentational account (SOMA) of consciousness. The theory unifies many other views and aptly paints a coherent picture of how a system like the human brain, can experience consciousness. In addition to SOMA, however, Attention Schema Theory of Consciousness (AST) also provides an account of both conscious experience and how a system comes to believe it has conscious experience, by combining different theories (Graziano, 2016, 2019). Despite the plenty of core features that are shared by the two views, here I would like to highlight a potential point of conflict, that I argue makes AST a better account of consciousness than SOMA, at least in its current form.

## How Consciousness is Defined in SOMA and in AST

SOMA treats consciousness as a higher-order network that acts as an observer of that systems' first-order information. This way, a system can be sensitive to its own internal states. Cleeremans et al. list three conditions that, in their account, phenomenal awareness would depend on:

(i) the existence of massive information-processing resources that are sufficiently powerful to simulate certain aspects of one's own physical basis and inner workings; (ii) the operation of continuously learning systems that attempt to predict future states; and (iii) immersion in a sufficiently rich social environment, specifically, environments from which models of yourself can be built. Which organisms meet these criteria is, obviously, an open and challenging empirical question. (Cleeremans et al., 2020a, p. 10)

This view, however compelling, seems at odds with evolutionary thinking. Not only does this theory imply that only social animals with a sophisticated brain may get the luxury of consciousness, but also it can lead some to subsequently conclude a binary nature for subjective experience [see Overgaard and Kirkeby-Hinrup (2020), Cleeremans et al., 2020b].

In the Attention Schema Theory of consciousness (AST), the “phenomenal experience” or the “what it is it likeness” is caused as the brain models its own attention. Graziano uses the analogy of a body schema, to argue for the existence of an attention schema (Graziano, 2016; Graziano et al., 2020). This is inspired by principles of control theory, in which having a model of something (here, attention) allows for exercising better control over it (Camacho and Bordons Alba, 2004). According to AST, we can claim consciousness by using our language apparatus when the information in the attention schema becomes accessible to higher-order systems in the brain (Graziano, 2020).

## Discussion: Why Consciousness Does Not Need a Social “Bedrock”

Cleeremans et al. (2020a) acknowledge the complex and loopy relationship between the theory of mind, perceptual awareness, and self-awareness. By taking this interdependent relationship into consideration, they add:

In this light, the social world is thus instrumental in generating conscious experience, for something special happens when we try to build a model of the internal, unobservable states of agents that are just like ourselves. (Cleeremans et al., 2020a, pp. 9-10)

When perceived from a top-down perspective, this is an excellent description of how the social world plays its role in generating phenomenal experience in the *human* brain. Given the complex interdependence of perceptual awareness, self-awareness, and the theory of mind systems, and given that they all play their part in the process of consciousness, the social world is indeed instrumental in generating *our* sophisticated phenomenal experience. The question is, what happens in a less sophisticated brain, possessing a less sophisticated version of the aforementioned systems? Are of these networks (or similar version of them) *equally* required to generate conscious experience, in the sense that the absence of one would remove the entire phenomenal experience, or could some of them (e.g., the theory of mind network) perform their important role mainly through the activity of others, in the sense that in their absence, a different and arguably less sophisticated phenomenal experience would still remain? This distinction is critical given that if the former is true, then there are strong reasons to believe that many animals are not conscious but simply *sensitive* to their surroundings, whereas should the latter be true, then a case can be made for a gradual *sophistication* of consciousness during the evolutionary history. Cleeremans et al. (2020b) indeed argue that at least a moderate version of SOMA could be compatible with animal consciousness; however, further elaboration on this area should be fruitful.

From an evolutionary stance, one can argue that a model of the “self” can gradually form or be *inferred*, for the primary reason of enforcing better control over the behavior of the organism, providing a “user- illusion” of the system to itself (Dennett, 2017; Hohwy and Michael, 2017). By the same token, a model of attention can also develop, for the system to better control how its cognitive resources are being spent. From that moment which an ancestral version of each network is in place, if the organism evolves in a social environment, these networks may get to be taxed for additional purposes over time (i.e., assigning traits and ultimately consciousness, to others). This predicts a model of others to overlap with a model of the self and seems in line with the existence of conditions such as mirror-touch synesthesia, in which one has a tactile subjective experience simply by observing touch to someone else's body (Banissy et al., 2011).

None of the aforementioned networks (the self and the attention-schema) however, seem to *necessitate* a social environment to initially develop, as they independently can provide evolutionary advantages to their bearer. However, being in a social environment over multiple generations may in fact influence how these networks interact and co-evolve. This view allows for a gradual appearance of consciousness which does not require a social environment to begin with and consequently, does not potentially exclude other animals from having phenomenal consciousness simply because they do not share a social history as humans do. As long as the system has attention, it can benefit from an attention schema and as long as it has an attention schema, it is conscious. This idea is well-compatible with AST, which in addition to providing an explanation for consciousness, also provides an evolutionary basis for it (Graziano, 2019).

In conclusion, if we are to argue for a functional role of consciousness, AST's account of phenomenal experience appears to be more a parsimonious one whereas SOMA's account, if it involves sociality as a necessary precondition, seems at odds with a gradual evolutionary view. Both frameworks, however, ultimately aim to explain why it is that we think we are conscious, rather than taking our “intuition” of subjective experience at face value. This idea has been advocated by Dennett (1993) and others (Frankish, 2016) and may be key in bringing us one step closer to a scientific account of conscious experience.

## Author Contributions

The author confirms being the sole contributor of this work and has approved it for publication.

## Conflict of Interest

The author declares that the research was conducted in the absence of any commercial or financial relationships that could be construed as a potential conflict of interest.
